# Breast Cancer With a HER2 IHC2+ and FISH HER2/CEP17 Ratio ≥2.0 and an Average HER2 Gene Copy Number <4.0 per Tumor Cell: HER2 mRNA Overexpression Is a Rare Event

**DOI:** 10.3389/fonc.2020.00985

**Published:** 2020-06-19

**Authors:** Yuanyuan Liu, Shafei Wu, Xiaohua Shi, Feng Mao, Xuan Zeng

**Affiliations:** ^1^Molecular Pathology Research Center, Department of Pathology, Peking Union Medical College Hospital, Chinese Academy of Medical Sciences, Beijing, China; ^2^Department of Breast Surgery, Peking Union Medical College Hospital, Chinese Academy of Medical Sciences, Beijing, China

**Keywords:** breast cancer, HER2, equivocal, copy number, ratio, mRNA

## Abstract

**Purpose:** For breast cancer, accurately illustrating HER2 characteristics is a critical precondition for evaluating the prognosis and predicting the efficacy of anti-HER2 therapy. Our purpose was to expose HER2 mRNA expression through an *in situ* hybridization assay (RNAscope), to aid the identification of HER2 status in breast cancers with a previously controversial classification for patients suffering from a HER2 IHC2+ and HER2/CEP17 ≥2.0 and a <4.0 mean HER2 gene copy number/cell (entitled FISH group 2 by update 2018 HER2 testing guideline).

**Methods:** A total of 8,983 cases of breast cancer with a known HER2 status detected by initial IHC, and a necessary reflex FISH assay for those with IHC2+, were retrospectively analyzed. Then, 41 cases of HER2 IHC2+ in the FISH group 2 were collected and a RNAscope was performed.

**Results:** The incidence of breast cancers with IHC2+ and in the FISH group 2 was 0.46% (41/8,983) in our single-institutional study cohort. In most of the cases (27/41, 65.9%), low levels of HER2 mRNA expression (score 1 and 2 by RNAscope) were demonstrated. Only one case (1/41, 2.4%) of high-level HER2 mRNA expression (score 4 by RNAscope), harboring a FISH HER2/CEP17 ratio of 2.06 and an average HER2 copy number of 3.70, was revealed. One case with the highest FISH HER2/CEP17 ratio of 3.90, showed the lowest level of HER2 mRNA expression (score 1 by RNAscope). Two cases with the same highest average HER2 signals/cell (3.95) by FISH possessed score 3 and score 2 with RNAscope, respectively. No cases with a score of 0 by RNAscope occurred in our sample. In the majority of cases (35/41, 85.4%), hypodisomy of chromosome 17 (average CEP17 signals/cell ≤1.75) was observed. There was no significant relationship between the mRNA expression and FISH results (average HER2 signals/cell, average CEP17 copy number, or HER2/CEP17 ratio) and clinicopathological features (ER and PR statuses, Ki 67 index, tumor size, and lymph node metastasis) in our population.

**Conclusion:** HER2 mRNA overexpression was not a feature in our group of patients. Based on our data, breast cancers with HER2 IHC2+ and in FISH group 2 support a categorization of HER2 negative.

## Introduction

Much data has shown that human epidermal growth factor receptor 2 (HER2) gene amplification and/or protein overexpression occurs in ~25 to 30% of all breast cancers and closely contributes to a poor prognosis as well as an encouragingly good response to HER2-targeting agents, such as anti-HER2 monoclonal antibodies and antibody–drug conjugates (e.g., trastuzumab, pertuzumab, and trastuzumab-emtansine) (1–3). Therefore, it is of crucial clinical importance to accurately identify the HER2 status of breast cancers.

Immunohistochemistry (IHC) and fluorescence *in situ* hybridization (FISH) are recommended for HER2 testing in breast cancers as per the guidelines from the American Society of Clinical Oncology (ASCO)/College of American Pathologists (CAP) and Breast Cancer Expert Panel of China (4, 5). In China, the IHC assay, which is of widespread popularity, is used for initial HER2 testing in breast cancer based on automatically staining a platform of Ventana Benchmark with 4B5 primary antibody, followed up by those well-acquainted with the interpretation criteria. Compared to IHC, FISH assay [commonly used as dual-probe including the HER2 gene and centromere enumeration probe for chromosome 17 (CEP17)], for inspecting HER2 gene copy numbers and amplification because it is more precise in recognizing a HER2 status based on its available research and clinical evidence, has been widely applied for reflex examination and confirmation of HER2 status in specimens with equivocal HER2 IHC results (IHC2+) (4). For most patients, HER2 status can be determined through IHC and if necessary, followed by FISH detection and vice versa (initial HER2 detection by FISH followed by IHC for FISH-equivocal cases). However, for a few cases with uncommon HER2 features and a very low incidence (<10%), the categorization of HER2 is still controversial, because there has been less data reported from clinical studies (4, 5).

According to the 2018 update of the ASCO/CAP HER2 testing guidelines, HER2 IHC3+ and IHC1+ or 0 are to be explicitly classified as HER2 positive and negative, respectively, when initial HER2 testing uses an IHC assay. Furthermore, cases with HER2 IHC2+ should be detected by FISH assay. Then, HER2 status can finally categorized as HER2 positive when the HER2/CEP17 ratio is ≥2.0 and the average HER2 gene copy number is ≥4.0 per tumor cell (group 1), HER2 status is considered as HER2 negative when the HER2/CEP17 ratio is <2.0 and the average HER2 gene copy number is <4.0 per tumor cell (group 5) by dual-probe FISH assay. However, HER2 status should be determined conversely depending on the IHC findings for the additional three subgroups of cases, which include HER2/CEP17 ratio ≥2.0 and an average HER2 gene copy number <4.0 per tumor cell (group 2), HER2/CEP17 ratio <2.0 and an average HER2 gene copy number ≥6.0 per tumor cell (group 3), and HER2/CEP17 ratio <2.0 and an average HER2 gene copy number >4.0 and <6.0 per tumor cell (group 4). With respect to cases of HER2 FISH group 2, the HER2 status should be assigned as HER2 positive when it is HER2 IHC3+, otherwise the HER2 status should be interpreted as HER2 negative when it is HER2 IHC2+, 1+, or 0, ultimately according to the 2018 guideline (4).

Looking back to the recommendation for HER2 classification on the 2013 version of the HER2 testing guidelines, although extra HER2 testing was suggested, cases with a HER2/CEP17 ratio ≥2.0 and an average HER2 copy number <4.0 per tumor cell (called group 2 by 2018 guideline) were automatically classified as HER2 positive regardless of the HER2 IHC results (including IHC2+ which should be further re-tested by FISH, but where IHC3+, 1+, or 0 would not be, usually). This was mainly based on evidence from the HERA trial, in which the patients with a <4.0 average HER2 gene copy number/cell (<10%) any reduced benefit from trastuzumab therapy was not observed, even though HER2 IHC findings were not provided (unknown proportion of cases with IHC2+ in the FISH group 2) (6). In contrast, according to the updated 2018 guidelines, cases with HER2 IHC2+ and in FISH group 2 are definitively considered as HER2 negative, which is basically dependent on the findings of two retrospective studies (BCIRG-005 for HER2 negative trial and BCIRG-006 for HER2 positive trial) in which there was no HER2 IHC3+ in the subgroup with a HER2/CEP17 ratio ≥2.0 and an average HER2 copy number <4.0 per tumor cell for 35 cases in whom IHC was performed, despite there only being three cases with IHC2+. Unfortunately, data on the efficacy of adjuvant trastuzumab treatment were not available due to the quite small number of cases, just like the HERA trial mentioned above (4, 7). Accordingly, the reported data were not sufficient for assigning such a small number of breast cancers with this unusual HER2 pattern to HER2 negative.

In the present study, a total of 8,983 formalin-fixed paraffin-embedded (FFPE) samples, with invasive breast cancers archived in our institution between Jan 2013 and Aug 2019, were retrospectively analyzed. Of these, 41 cases were recorded as HER2 FISH group 2 with HER2 IHC2+. Then, HER2 mRNA expression was subsequently assessed by RNA in situ hybridization (a technique named RNAscope, the feasibility for HER2 test in breast cancer was validated by our previous studies), which is a visualization method combining molecular signals and an almost intact morphological information of tissues and cells containing natural internal control (non-cancerous area), similar to FISH and IHC (8–10). Our aim was to explore a molecular cytogenetic basis for categorizing HER2 statuses in breast cancer with HER2 IHC2+ and FISH group 2.

## Materials and Methods

### Patients and Specimens

**Figure 1:**
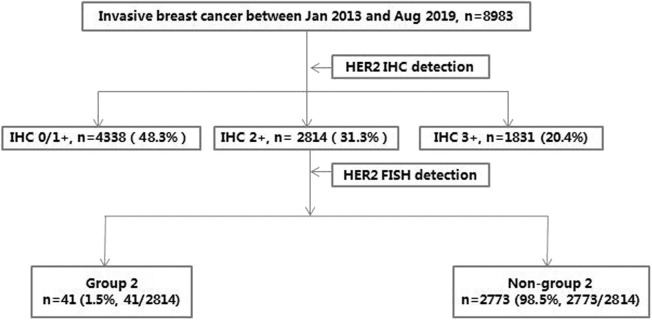
The current study algorithm.

A total of 8,983 consecutive cases from invasive breast cancers, involving 41 cases of HER2 IHC2+ and FISH group 2 archived in Peking Union Medical College Hospital between Jan 2013 and Aug 2019, were retrospectively analyzed. The HER2 status was initially detected by IHC for all samples and then by FISH for IHC2+ samples, along with conventional pathological diagnosis. For the 41 cases of HER2 IHC2+ and FISH group 2 (37 cases of primary lesion and 4 cases of recurrent lesion), HER2 mRNA transcript levels were further investigated using FFPE samples by RNAscope, the algorithm for this cohort is presented in Figure 1. Disease-free survival (DFS) was calculated (follow-up ended on December 31, 2019).

### IHC

IHC was conducted on a 4 μm thickness FFPE tissue section for HER2 protein expression evaluation based on the Ventana Benchmark Ultra autostainer platform (Ventana Medical Systems, Inc., Tucson, AZ, USA), with an antibody of clone 4B5 according to the manufacturer's protocol. IHC slide was scored blind by two independent pathologists. HER2 expression were interpreted according to the 2013 ASCO/CAP HER2 testing guidelines for the samples archived between 2013 and 2018, and according to the 2018 ASCO/CAP HER2 testing guidelines for those obtained in 2019. HER2 was classified as IHC 0, 1+, 2+, or 3+. IHC3+ was defined as HER2 positive, while IHC1+ and IHC0 are considered as HER2 negative, and IHC2+ is HER2-equivocal.

### FISH

FISH assays were performed on a 4 μm thickness FFPE slide using a PathVysion HER2 DNA probe kit (Vysis/Abbott, Abbott Park, Illinois), based on the Thermo-Brite Elite automated FISH slides prep system (Leica, Richmond, CA, USA) and following the standard operating manual. A HER2 gene with red color (labeled by SpectrumOrange) and a CEP17 with green color (labeled by SpectrumGreen) were counted, respectively in more than 20 nuclei from at least two areas of invasive tumor via the CytoVision DM6000B fluorescent microscope system (Leica, Biosystem, Buffalo Grove, IL). FISH slide was recounted by an additional observer who blinded to previous FISH results for ambiguous cases. When the ratio of HER2/CEP17 was ≥2.0 or the average HER2 gene copy number/tumor cell was ≥6.0 with a ratio of HER2/CEP17 <2.0, the HER2 status was classified as positive. When the ratio of HER2/CEP17 was <2.0 with an average HER2 signals/cell <4.0, the HER2 status was considered negative. When the ratio of HER2/CEP17 was <2.0 with an average HER2 signals/cell of ≥4.0 and <6.0, the HER2 status was regarded as equivocal, according to the 2013 guidelines, or positive (if IHC3+) and negative (if IHC0, 1+, or 2+) according to the 2018 guidelines, respectively. When the ratio of HER2/CEP17 was ≥2.0 and the average HER2 signals/cell were <4.0, the HER2 status was defined as positive according to the 2013 guidelines, or positive (if IHC3+) and negative (if IHC0, 1+, or 2+) according to the 2018 ASCO/CAP guidelines, respectively.

### RNAscope

RNAscope was carried out on three 4 μm FFPE slides, one for HER2 mRNA testing with a HER2 probe pool (20 probe pair set which targeting HER2 mRNA sequence), one for positive control with a housekeeping gene PPIB (peptidylprolyl isomerase B) probe (to evaluate RNA integrity), and one for negative control with a bacterial gene DapB probe (to evaluate background staining) (Advanced Cell Diagnostics, Inc., Hayward, CA). The HER2 mRNA signals were detected and amplified using the RNAscope FFPE 2.5 kit, according to the testing instruction guidelines described previously (8–11). In brief, the FFPE slide was treated with a citrate buffer and protease solution at 40°C for 30 min after dewaxing and followed by hybridization with probes at 40°C for 2 h. The specific signal was amplificated using a preamplifier and amplifier reagents and was then detected by DAB staining and counterstaining via hematoxylin sequentially. The whole slides were scanned using an Aperio CS2 digital image system under 40 X objective lens (Leica Biosystems, San Diego, CA, USA). At least 100 tumor cells were scored. HER2 mRNA expression was categorized in five degrees from low to high: Score 0 (<1 average HER2 signals/tumor cell), score 1 (average 1–3 of HER2 signals/tumor cell), score 2 (average 4–9 HER2 signals/tumor cell, with or without a few cluster signals), score 3 (average 10–15 HER2 signals/tumor cell, or cluster signals were observed in <10% tumor cells), and score 4 (>15 average HER2 signals/tumor cell, or cluster signals were observed in ≥10% tumor cells).

### Statistical Analysis

Statistical analyses were conducted using SPSS version 22.0 and GraphPad Prism 8.0. Comparisons of the clinicopathological characteristics and RNAscope results were performed using Kruskal–Wallis H test. Spearman rank correlation test was used to analyze the relationship of HER2 mRNA expression by RNAscope and HER2 FISH results. Survival statues were calculated using the Kaplan-Meier method and compared via log-rank test. *P*-value of < 0.05 was defined as significant.

## Results

**Figure 2:**
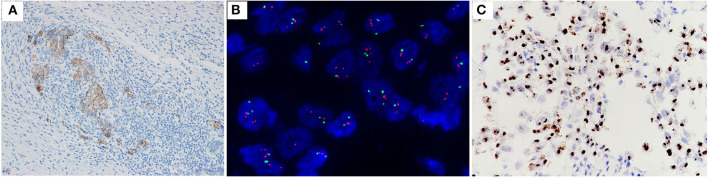
Case no. 31, with IHC2+ **(A)**, a HER2/CEP17 ratio of 2.06, and an average HER2 signals/cell of 3.70 **(B)**. Showed high level of HER2 mRNA expression (score 4 by RNAscope, with a cluster signals pattern in tumor, and without any signal in non-tumor cells) **(C)**.

**Figure 3:**
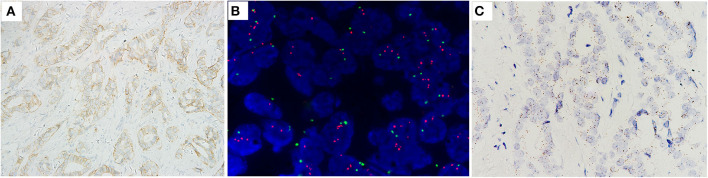
Case no. 19, with IHC2+ **(A)**, a HER2/CEP17 ratio of 2.26, and the highest average of HER2 signals/cell of 3.95 **(B)**. Showed low level of HER2 mRNA expression (score 2 by RNAscope) **(C)**.

**Figure 4:**
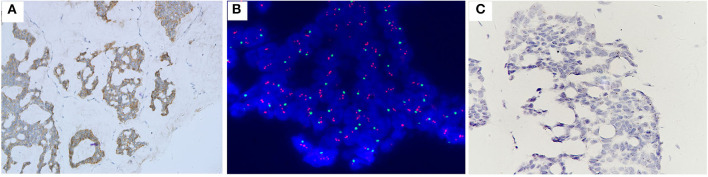
Case no. 30, with IHC2+ **(A)**, the highest HER2/CEP17 ratio of 3.90, the lowest average CEP17 copy number/cell of 1.00, and average HER2 signals/cell of 3.90 **(B)**. Showed the lowest level of HER2 mRNA expression (score 1 by RNAscope) **(C)**.

**Figure 5:**
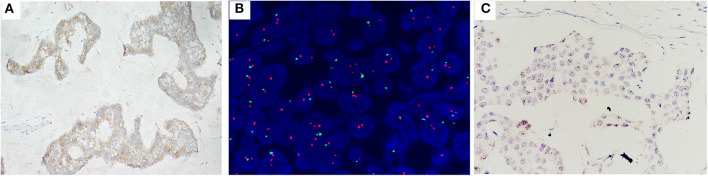
Case no. 8 with, IHC2+ **(A)**, the lowest HER2/CEP17 ratio of 2.00, and the lowest average HER2 signals/cell of 2.10 **(B)**. Showed the lowest level of HER2 mRNA expression (score 1 by RNAscope) **(C)**.

The frequency of breast cancers with IHC2+ and FISH group 2 was 0.46% (41/8,983) in our study population. The age of the patients was between 28 and 79 years old, including 38 cases of non-special-type invasive cancer, one case of invasive micropapillary cancer (case no. 10), and two cases of mucinous cancer (case nos. 8, 30), of which 18 cases had lymph node metastases. In our cohort, one case had a score of 4, 13 cases a score of 3, 15 cases a score of 2, and 12 cases a score of 1 by RNAscope. None of the cases with a score of 0 by RNAscope are shown. Case nos. 1 and 19, with the highest average HER2 gene copy number per tumor cell (3.95) by FISH, harbored scores of 3 and 2 by RNAscope, respectively. Case no. 8, with the lowest average HER2 signals/cell (2.10), displayed a score of 1 by RNAscope, and also had the lowest HER2/CEP17 ratio of 2.00 in the study group. Case no. 30, with the highest HER2/CEP17 ratio of 3.90, exhibited a score of 1 by RNAscope. Case no. 31, with the highest level of mRNA expression (score 4 by RNAscope), had cluster HER2 transcript signal features showing a HER2/CEP17 ratio of 2.06 and 40% of Ki 67 index. However, case no. 17 and 36, with the same levels of HER2 RNA expression (score 3 by RNAscope), possessed the highest (90%) and lowest (5%) Ki 67 indexes, respectively. The lowest HER2 mRNA expression with a score of 1 by RNAscope, was found in 12 cases, including case nos. 6, 8, 12, 16, 21, 22, 23, 27, 29, 30, 33, and 34, with the average HER2 signals/cell being 3.90, 2.10, 3.20, 3.65, 3.70, 3.40, 3.93, 3.55, 2.25, 3.90, 3.48, and 3.70, respectively. Case no. 34, with the highest average CEP17 copy numbers per tumor cell (1.83), observed the lowest HER2 mRNA levels (score 1 by RNAscope). The lowest HER2 mRNA level (score 1 by RNAscope) with lowest average CEP17 copy number/cell (1.00), occurred in case no. 30. The great majority of cases (35/41, 85.4%) in our cohort belonged to hypodisomy, according to the criteria for chromosome 17 enumeration as described previously (average CEP17 signals/cell ≤1.75, 1.76–2.25, and ≥2.26 were classified as hypodisomy, disomy, and polysomy respectively) (12) (Figures 2–5).

**Figure 6:**
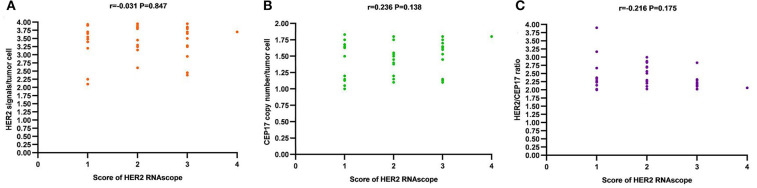
The correlation of HER2 mRNA expression by RNAscope (score 1 to 4) and HER2 FISH features [average HER2 signals/tumor cell **(A)**, average CEP17 copy number/tumor cell **(B)**, and HER2/CEP17 ratio **(C)**].

Low expressions of HER2 mRNA with a score of 1 or 2 by RNAscope (27/41, 65.9%) were the predominate trait, and high-level HER2 mRNA overexpression with a score of 4 by RNAscope (1/41, 2.4%) was a rare event in our cohort. A relationship between the average HER2 gene copy number per tumor cell, average CEP17 copy number, and HER2/CEP17 ratio and the level of HER2 mRNA expression was not observed (Figure 6). The correlation between the level of HER2 mRNA expression by RNAscope and clinicopathological features, including ER and PR statuses, Ki 67 index, tumor size and lymph node metastasis, was not found (Table 1).

**Table 1:** Comparison of HER2 mRNA expression and clinicopathological characteristics of breast cancers with IHC2+ and FISH group 2 in the study cohort.

**Characteristics**	**HER2 RNAscope Results (score)**				***P***
	**1 (*n* = 12)**	**2 (*n* = 15)**	**3 (*n* = 13)**	**4 (*n* = 1)**	
**Age(years)**					
Mean (range)	49.33 (28~78)	44.07 (33~58)	56.31 (30~79)	61	
**Histopathological types**					
Non-special-type invasive cancer	10	14	13	1	0.458
Special-type invasive cancer	2	1	0	0	
**Ki 67 index**					
<14%	2	4	4	0	0.798
≥14%	10	11	9	1	
**Tumor size**					
≤ 2.0 cm	7	7	11	0	0.122
>2.0 cm	5	8	2	1	
**Lymph node**					
Negative	8	7	8	0	0.483
Positive	4	8	5	1	
**ER status**					
Negative	2	2	3	1	0.208
Positive	10	13	10	0	
**PR status**					
Negative	4	2	5	1	0.190
Positive	8	13	8	0	

**Figure 7:**
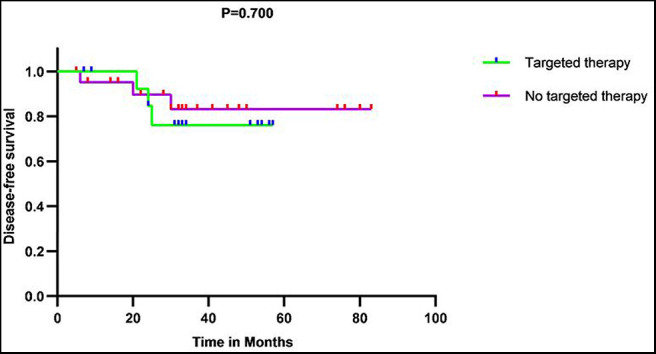
Disease-free survival (DFS) curves showed there was no significant DFS difference between patients with and without targeted therapy in 37 patients with primary tumor in our series.

Fifteen patients with primary tumors and three patients with recurrent tumors were treated with trastuzumab in our cohort. The survival analyses showed there was no statistically significant DFS difference for patients with or without targeted therapy (Figure 7).

## Discussion

According to the updated 2018 HER2 testing guidelines, compared with HER2 FISH group 3 and group 4, which are currently recognized as HER2 positive and negative respectively, the much rarer FISH group 2, particularly IHC-equivocal (2+) cases within the group, were so exceptionally infrequent that it was extremely difficult to assign a HER2 status due to a lack of ample evidence. There is controversy in HER2 judgement when a case with IHC2+ and a FISH HER2/CEP17 ratio of ≥2.0 and <4.0 of average HER2 signals/cell which already described in 2013 version guideline on opposite views. Consequentially, for this subset of patients, prognosis evaluation and efficacy predictions of disease are challenging, and the study was meaningful for accumulating valuable data.

Zare et al. analyzed 1,201 consecutive cases with breast cancer. Overall, 18 out of the 1,201 samples were declared to have a FISH HER2/CEP17 ratio ≥2.0 and an <4.0 average HER2 signal/cell, of which there were seven cases with HER2 IHC2+. The frequency of cases with HER2 IHC2+ and FISH group 2 was 0.58% (7/1,201), without more information provided on the basis of a limited number of specimens (13). Moreover, the frequency of HER2 IHC2+ and FISH group 2 was 0.81% (65/8,068) in a multi-center study (14). Our single-institutional study indicated that the rate of breast cancers with HER2 IHC2+ and FISH group 2 was 0.46% (41/8,983), which is slightly lower than previously published data.

Amongst the 41 cases with HER2 IHC2+ and of FISH group 2 in our cohort, 35 cases had a ≤1.75 average CEP17 copy number/cell by FISH, i.e., hypodisomy, which was ascribed to a loss of the whole chromosome or a partial deletion of chromosome 17 involving the centromere for the FISH result to be increased to a HER2/CEP17 ratio of 2.0 or greater, rather than a “true” HER2 gene amplification as represented in literature (14, 15). Thus, HER2 FISH group 2 was also sometimes directly known as “monosomy” (14). However, this was not sufficient for determining these cases as HER2 negative only, depending on limited available evidence of chromosome 17 phenotype combined with a HER2/CEP17 ratio by FISH, because the moderate expression of the HER2 protein (IHC2+) existed in all of the cases, for which the mechanism needs to be elucidated. Therefore, more evidence has to be sought to support HER2 classification.

Most of the previous data related to therapies supporting the determination of HER2 status in breast cancers are largely based on responsiveness to treatment with the anti-HER2 targeted drug trastuzumab. As known to all, a number of patients who are HER2 positive still inevitably show a poor response to trastuzumab (blocking HER2/HER2 homodimer primarily and disturbing the dimerization resulting from HER2 and additional HER family members). In recent years, several new therapeutic strategies, including pertuzumab (mainly inhibiting the HER2/HER3 heterodimer) and lapatinib (in which there is a lack of extracellular anti-HER2 binding domain p95 HER2 sensitive) were developed for enhancing the activity of trastuzumab and improving disease control (3). Accordingly, HER2 classifications assisted by the therapeutic benefit of trastuzumab are also insufficient. In this context, it was very significant for further disclosing the characteristics of the HER2 molecule in breast cancer with IHC2+ and FISH group 2.

We analyzed HER2 mRNA expression by RNAscope for 41 cases of breast cancer with HER2 IHC2+, and the average HER2 signals/cell was **<**4.0 with a HER2/CEP17 ≥2.0. Only one case (1/41, 2.4%) with a high-level expression of HER2 mRNA (score 4 by RNAscope) was distinguished. Most of the cases (27/41, 65.9%) harbored low-level expressions of HER2 mRNA (score 1 or 2 by RNAscope). Hence, our findings imply that HER2 mRNA overexpression does not occur frequently in this subtype and supports the recommendation of the 2018 HER2 testing guidelines to classify breast cancers with HER2 IHC2+ and FISH group 2 as HER2 negative. In addition, there was no cases with a score of 0 by RNAscope, so a moderate expression of the HER2 protein (IHC2+) was of real existence rather than a technique issue. The mechanisms behind this needs to be disclosed in the future. Besides, there was no association between the average HER2 gene copy number per tumor cell and HER2/CEP17 ratio with HER2 mRNA expression in our study. The level of HER2 mRNA expression was quite different in our cases, which had the same level of HER2 protein expression (IHC2+), the impacts from different approaches which were aimed at HER2 protein and mRNA detection respectively, inherent performance of the two molecules, sample pretreatment and preparation referring to protein and mRNA, and unsuspected factors, were not excluded.

On the other hand, several investigations related to HER2 activation with a very low incidence (2%), in another way to breast cancer, which was absent of HER2 amplification by FISH and/or protein overexpression by IHC, showed HER2 gene mutations (such as V777L, L755S, D769H, V842I, G309A, etc.) (16, 17). Fortunately, cell proliferation and carcinogenic growth uncontrollably caused by HER2 gene mutations can be effectively suppressed with a regimen of special HER2-directed agents (e.g., neratinib), providing an additional treatment strategy for breast cancer (18, 19). Previous studies have exhibited HER2 gene mutations in breast cancers with IHC2+ and low HER2 gene copy numbers (16). For further exploring the cause of HER2 mRNA overexpression in our cohort, case nos. 18, 31, and 41 were analyzed by next generation sequencing (NGS). However, known pathogenic HER2 gene mutations were not discriminated (data not shown). The mechanisms behind the high expression of HER2 mRNA in rare case remains to be investigated. In addition, the cases with score 4 by RNAscope were all confirmed as HER2 positive in our previous investigations (9, 10), so score 4 was considered as high-level mRNA expression in the current study and could be one of interpretation criteria of HER2 positive for the cases with HER2 double-equivocal detected by IHC and FISH. The biological significance of score 3, moderate level of mRNA expression, probably was true HER2 equivocal in this group of patients, should be further inspected in the future. The stained slide by RNAscope retained intact morphological information which quite similar to the standard HER2 assessment assay (FISH or IHC). Therefore, RNAscope is an optimal approach for HER2 mRNA analysis. As the technology updates, the costs reduction and clinical applications are potentially possible even though it cannot be used for conventional diagnosis until it is clinically effectiveness validated. In conclusion, based on our findings, we suggest and support the judgement of breast cancers with IHC2+ and FISH group 2 as HER2 negative.

## Data Availability Statement

The raw data supporting the conclusions of this article will be made available by the authors, without undue reservation.

## Ethics Statement

The retrospective studies involving human participants were reviewed and approved by the institutional review board of Peking Union Medical College Hospital. The ethics committee waived the requirement of written informed consent for participation.

## Author Contributions

YL and SW performed the experiments. YL and FM collected the clinicopathological information. XS and YL reviewed the slides and analyzed the data. XZ designed the study and wrote the manuscript. All authors approved the submitted version of the manuscript.

## Conflict of Interest

The authors declare that the research was conducted in the absence of any commercial or financial relationships that could be construed as a potential conflict of interest.
